# Thoracic Involvement in Systemic Autoimmune Rheumatic Diseases: Pathogenesis and Management

**DOI:** 10.1007/s12016-022-08926-0

**Published:** 2022-03-18

**Authors:** Elena De Zorzi, Paolo Spagnolo, Elisabetta Cocconcelli, Elisabetta Balestro, Luca Iaccarino, Mariele Gatto, Francesco Benvenuti, Nicol Bernardinello, Andrea Doria, Toby M. Maher, Elisabetta Zanatta

**Affiliations:** 1 Respiratory Disease Unit, Department of Cardiac, Thoracic, Vascular Sciences and Public Health, Padova University Hospital, Padova, Italy; 2 Department of Medicine-DIMED, Padova University Hospital, Padova, Italy; 3 Keck School of Medicine University of Southern California, Los Angeles California, USA; 4 Interstitial Lung Disease Unit, Royal Brompton Hospital, London, UK; 5 National Heart and Lung Institute, Imperial College, London, UK

**Keywords:** Connective tissue diseases, Autoimmune rheumatic disorders, Interstitial lung disease, Pulmonary arterial hypertension, Pleuritis, Airways disease

## Abstract

Thoracic involvement is one of the main determinants of morbidity and mortality in patients with autoimmune rheumatic diseases (ARDs), with different prevalence and manifestations according to the underlying disease. Interstitial lung disease (ILD) is the most common pulmonary complication, particularly in patients with systemic sclerosis (SSc), idiopathic inflammatory myopathies (IIMs) and rheumatoid arthritis (RA). Other thoracic manifestations include pulmonary arterial hypertension (PAH), mostly in patients with SSc, airway disease, mainly in RA, and pleural involvement, which is common in systemic lupus erythematosus and RA, but rare in other ARDs.

In this review, we summarize and critically discuss the current knowledge on thoracic involvement in ARDs, with emphasis on disease pathogenesis and management. Immunosuppression is the mainstay of therapy, particularly for ARDs-ILD, but it should be reserved to patients with clinically significant disease or at risk of progressive disease. Therefore, a thorough, multidisciplinary assessment to determine disease activity and degree of impairment is required to optimize patient management. Nevertheless, the management of thoracic involvement—particularly ILD—is challenging due to the heterogeneity of disease pathogenesis, the variety of patterns of interstitial pneumonia and the paucity of randomized controlled clinical trials of pharmacological intervention. Further studies are needed to better understand the pathogenesis of these conditions, which in turn is instrumental to the development of more efficacious therapies.

## Introduction

Autoimmune rheumatic diseases (ARDs) represent a large and heterogeneous group of systemic disorders characterized by inflammation and dysregulation of the immune system leading to tissue damage and fibrosis [1]. The thorax is the most frequently affected anatomical area, and lung involvement—in particular interstitial lung disease (ILD), pleural involvement and pulmonary arterial hypertension (PAH)—is present in a substantial proportion of patients with ARDs. In fact, ILD is found in up to 50% of patients with systemic sclerosis (SSc) and inflammatory idiopathic myopathies (IIMs), with variable percentages based on the diagnostic method employed in rheumatoid arthritis (RA, 4–48%), Sjögren’s syndrome (SS, 2–25%) and systemic lupus erythematosus (SLE, 3–13%) [2]–[4]. Pleural involvement is common in SLE and RA with up to 50% and 20% of patients, respectively, experiencing a history of pleurisy, but rare in other ARDs, whereas airway disease of both the upper and lower respiratory tract is mostly found in RA (39–60% of patients as assessed by chest high-resolution CT [HRCT]) [5]–[7]. In SLE and SSc, a significant minority of patients (1–5% and % 8–12%, respectively) develop PAH, a severe complication characterized by vascular remodelling of small pulmonary arteries [8].

The pathogenesis of thoracic involvement in ARDs is largely unknown, but is likely to involve a complex interplay between host/genetic and environmental factors, leading to chronic inflammation, endothelial dysfunction and fibrosis. However, different pathogenetic mechanisms may be involved based on the affected organ and the underlying disease. ILD is a leading cause of death in patients with SSc, IIMs and RA; the presence of PAH is also associated with poor prognosis in SSc, although recent data suggest that specific treatment improves outcomes [9].

Yet, the management of thoracic involvement in ARDs is based on a small number of randomized controlled trials (RCTs), observational studies or even expert opinion. In this regard, a better knowledge and understanding of the pathogenic mechanisms underlying thoracic involvement in ARDs is instrumental to the development of more efficacious therapies. In addition, a more precise definition of clinical presentation, disease course and response to treatment is of utmost importance in the management of these patients, for both rheumatologists and pulmonologists.

In this review, we summarize the current knowledge on lung involvement in ARDs, with emphasis on the pathogenesis of ILD, airways, pleural and vascular involvement, and explore current and future therapeutic strategies.

## Lung Parenchyma

ILD is the most common and severe form of lung involvement in ARDs, leading to significant morbidity and mortality. The highest prevalence of ILD is found in SSc, IIMs and RA [7].

**Fig. 1:**
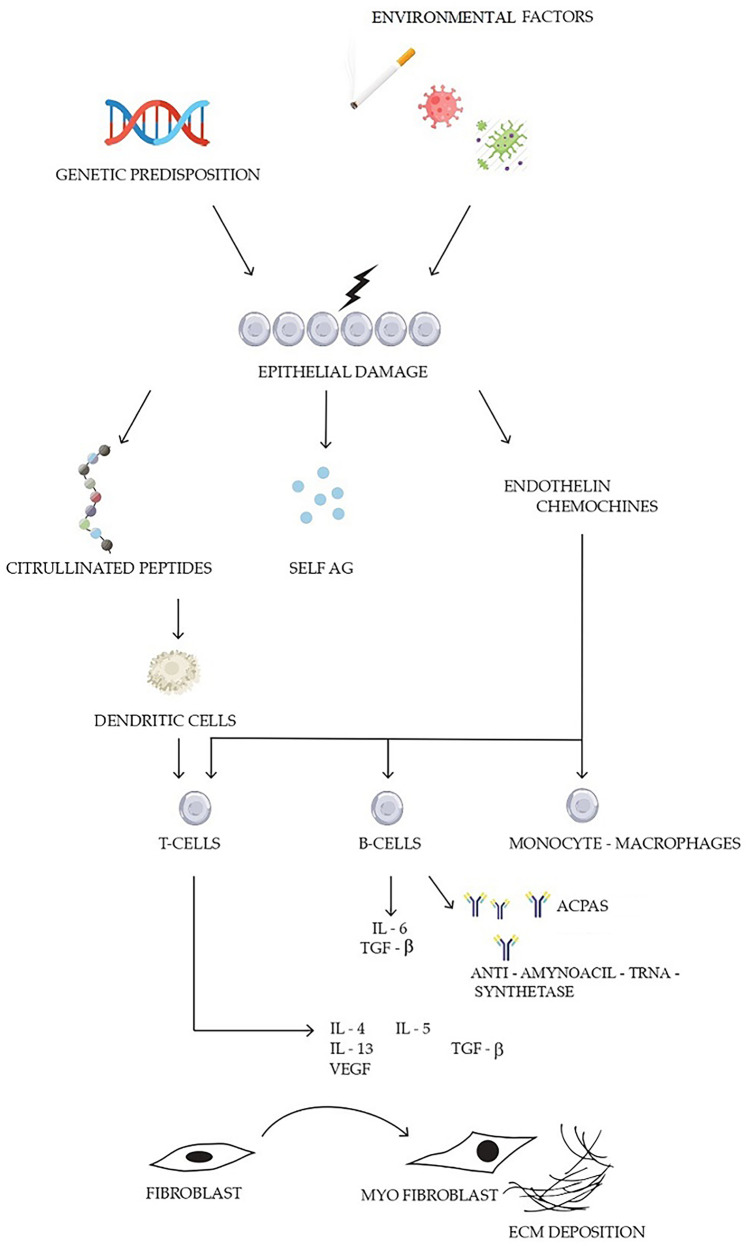
A pathogenetic model of parenchymal lung involvement in autoimmune rheumatic diseases. Environmental factors (cigarette smoke, pollutants and pathogens) in a genetically predisposed individual determine alveolar epithelial injury. This process results in damaged lung tissue, subsequent endothelial activation (in particular in systemic sclerosis) and release of self-antigens, which in turn lead to breakdown of self-tolerance and activation of the immune response. Multiple cellular compartments are involved: B-cells with production of autoantibodies, T-cells and monocytes–macrophages. Immune activation leads to the production of several cytokines such as IL-4, IL-5, IL-6, IL-13, TGF-β, TNF-α and VEGF that contribute to the initiation of repair pathways, including recruitment of fibroblasts and myofibroblasts. (ACPAs: anti-cyclic citrullinated peptide antibodies TGF-β: Transforming Growth Factor β, TNF-α: Tumor Necrosis Factor α, VEGF: Vascular Endothelial Growth Factor)

The pathogenesis of ARDs-ILD involves multiple compartments and the interaction between various cellular components, leading to the development of fibrosis and progression of lung damage. The key pathogenetic mechanisms involved in the development of ILD in ARDs are summarized in Fig. 1.

### Systemic Sclerosis

ILD is a major determinant of mortality in SSc. Data analysis from the European Scleroderma Trials and Research (EUSTAR) cohort that included 5,850 SSc patients revealed that of all deaths directly attributed to SSc, 35% were due to pulmonary fibrosis and 26% to PAH [10]. ILD is more frequent in patients with the diffuse cutaneous form of disease, compared to patients with limited cutaneous disease (60–70% vs. 35%) and in those with anti-topoisomerase I antibody positivity (50–80%) [11]. However, physicians should be aware that patients with both forms of skin involvement may develop ILD; accordingly, experts agree that all patients should be screened for ILD using HRCT at SSc diagnosis [12, 13]. Other risk factors associated with the development of SSc-ILD include male sex, African-American ethnicity, older age at disease onset and shorter disease duration [12]. In addition, there is growing interest in the identification of biomarkers of SSc-ILD, such as CCL18, squamous cell carcinoma antigen 1 (SCCA1) and Krebs von den Lungen 6 glycoprotein (KL-6) [14, 15].

Vascular injury, inflammation and activation of the immune system are the first alterations in the pathogenesis of SSc-ILD, resulting in lung fibrosis already in the early stages of the disease. Microvascular injury and subsequent endothelial cell activation induce endothelin-1 and chemokines production and increased expression of adhesion molecules, leading to the recruitment of a plethora of inflammatory cells [16]. Immune dysfunction is one of the most important contributors to the pathogenesis of SSc-ILD. In this regard, there is increasing evidence that immune activation is a cause and not a consequence of vasculopathy and fibrosis [17]. Among inflammatory cells, B-cells are thought to play a crucial role in SSc, as they show signs of hyperactivation; specifically, autoantibody production is increased, but polyclonal B-cell hyperactivity and hyper-γ-globulinaemia have also been described [18]. In patients with SSc, B-cells (both naive and memory B cells) overexpress CD19, a powerful positive regulator of their activity [19]. By contrast, there is evidence of impairment of the inhibitory CD22 receptor, probably due to the presence of serum anti-CD22 antibodies [18]. As previously mentioned, hyperactivation of B-cells leads to overproduction of autoantibodies, some of which (i.e. functional antibodies) promote a pro-inflammatory and/or a fibrotic response [20]. In fact, B-cells are also involved in the pathogenesis of fibrosis, mainly by secreting IL-6, which induces a Th2-dominant immune response. Th2 cytokines (i.e. IL-4, IL-5, IL-6 and IL-13) increase the deposition of extracellular matrix proteins, whereas Th1 cytokines, such as interferon-γ and IL-2, inhibit extracellular matrix deposition [19, 21]. Furthermore, activated B cells also secrete high amounts of transforming growth factor (TGF)-β, which is one of the main profibrotic cytokines [21].

T cells are also involved in the pathogenesis of SSc and their presence in affected tissues is believed to precede fibrosis. In fact, in skin biopsies from patients with SSc, infiltrates consisting of T cells and macrophages are found early, before any microscopic evidence of fibrosis. Furthermore, T cells with a memory phenotype are found in lung biopsy specimens from patients with SSc-ILD [22]. These cells are predominantly CD4+ and display markers of activation, with a prevalent Th2 cytokine profile [17, 22]. In SSc, a defective control of T cell activation by Tregs leads to T cell proliferation and cytokine secretion; indeed, several studies have shown numerical and functional changes (i.e. defective suppressive activity) of the Treg population, leading to loss of tolerance [17]. A Treg/Th17 imbalance has also been hypothesized to play an important pathogenetic role in SSc [23].

Alterations in macrophage polarization have been recently recognized among the key immune system abnormalities involved in SSc pathogenesis. Indeed, emerging evidence suggests a possible pathogenetic role of tissue macrophages that display an alternatively activated M2 phenotype. M1 macrophages trigger an intensive inflammatory response and tissue damage, whereas M2 are closely associated with Th2 response, tissue repair and fibrosis. A recent study reported higher percentages of circulating monocytes/macrophages co-expressing M1 and M2 surface markers in SSc patients compared to healthy subjects [24]. Levels of soluble CD163 (a marker of M2 macrophages) are elevated in the serum of SSc patients and on macrophages of affected skin and lung [16]. Moreover, a high percentage of circulating cells belonging to the monocyte/macrophage lineage and expressing surface markers of both M1 and M2 phenotypes has been found in the serum of patients with SSc-ILD [25].

**Fig. 2:**
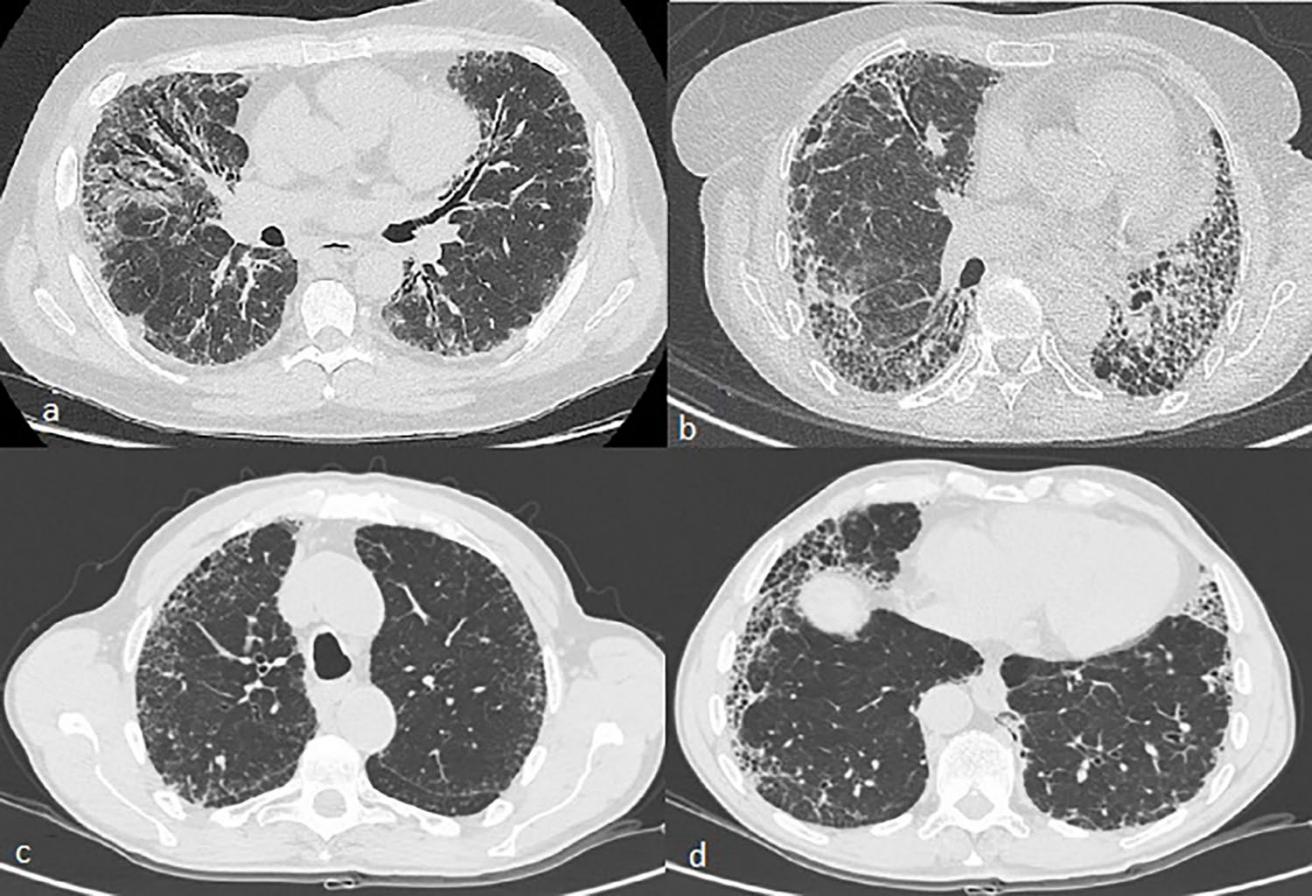
HRCT images. **a**: Fibrosing NSIP pattern in a patient with SSc. **b**: UIP pattern in a patient with overlap SSc-RA; **c** and **d**: UIP pattern in a patient with RA: HRCT shows reticular opacities and subpleural honeycombing

The most frequent disease pattern is non-specific interstitial pneumonia (NSIP), followed by usual interstitial pneumonia (UIP), which may be more frequently detected in patients affected by an overlap syndrome between SSc and RA [26] (Fig. 2). Ground glass opacities typical of NSIP rarely reverse with treatment, suggesting they might represent fine fibrosis rather than alveolitis; this is consistent with the observation that “fibrotic” NSIP is more common than “cellular” NSIP in SSc [7].

Pulmonary function tests (PFTs) tend to show a restrictive ventilatory defect, generally associated with a reduced diffusing capacity of the lung for carbon monoxide (DL_CO_), even in the absence of symptoms [12]. Reduced DL_CO_ is a highly sensitive and early indicator of possible ILD; however, it is not specific as it can also be reduced in SSc patients with PAH [27] or concomitant emphysema. Moreover, a moderate, isolated reduction of DL_CO_ (55–80% of the predicted values) is a common finding in patients with SSc and may be also due to pulmonary microangiopathy and/or subclinical alveolitis [27]. On the other hand, a restrictive ventilatory defect may be present in the absence of lung involvement as a consequence of extra-parenchymal impairment due to severe chest wall tightening from skin fibrosis in diffuse cutaneous SSc [28]. Although HRCT shows signs of parenchymal involvement in up to 90% of patients with SSc, less than half (45%) develop a moderate to severe ILD and only 13% have a severe impairment of lung function (i.e. forced vital capacity (FVC) < 50%) [29, 30]. Indeed, most SSc-ILD patients experience a slow decline in lung function, with only a minority of them progressing rapidly after ILD onset [12]. The main factors associated with ILD progression include early onset of ILD (i.e. within 3 years after SSc diagnosis), anti-topoisomerase I antibody positivity, presence of arthritis and tendon friction rubs [12, 31]. Moreover, extensive fibrosis on HRCT (i.e. > 20% of the total lung volume), reduced FVC and DL_CO_ at ILD diagnosis, significant decline in FVC or DL_CO_ after one year (more than 10% and 15%, respectively) and reduction of DL_CO_ over 3 years are associated with progression to severe ILD and increased mortality [12, 31, 32]. Once ILD has been identified, there is no consensus among experts regarding monitoring of lung disease with HRCT. According to clinical practice of expert centres, HRCT should be repeated initially every 6–12 months in association with PFTs every 4 to 6 months, in order to identify rapidly progressive forms [33]. A further challenge is represented by patients without ILD at diagnosis, for whom the exact timing for radiological and functional assessment has not been established. In common practice, PFTs are obtained yearly in patients with SSc [33].

### Rheumatoid Arthritis

The prevalence of clinically significant ILD in patients with RA is around 10%, rising to 58% if subclinical lung involvement is considered [7]. Older age at diagnosis, male sex, a history of ever smoking and rheumatoid factor (RF) or anti-cyclic citrullinated peptide antibody (ACPA) positivity are well known risk factors for the development of ILD in patients with RA [7, 34–36]. Genetic and environmental factors are also thought to play an important role in the development of RA-ILD. In particular, HLA–B54, HLA–DQB1*0601, HLA–B40 and the site encoding α-1 protease inhibitor are associated with an increased risk of ILD in patients with RA [3].

Evidence suggests that the initiating events that precede clinically manifest RA might originate at one or more mucosal sites, in particular the airways and the lung, as a consequence of environmental exposure, especially smoking [37]. Indeed, smoking is a risk factor not only for RA but also for RA-ILD, as it determines lung injury that contributes to the citrullination of proteins and activates profibrotic pathways [3, 38]. Persistent or repetitive injury to the airway mucosa or distal lung structures leads to increased protein citrullination and activates the innate immune system leading to an inflammatory response. In susceptible individuals, immune tolerance breaking causes an autoimmune response with further production of anti-citrullinated protein antibodies (Fig. 1) [39].

ACPAs, RF and other RA-related antibodies can be found in the serum of patients some years before any evidence of inflammation and immune reaction in the joints. RA-related autoantibodies have been identified also in induced sputum of at-risk seropositive individuals, patients with early-stage RA as well as in seronegative individuals at risk of developing RA [37, 39, 40]. In addition, an increased number of citrullinated peptides was found in the lung parenchyma of patients with RA-ILD, suggesting that the lung might locally produce RA-related autoimmunity [41]. These findings support the hypothesis that the lung itself might be the initial site of immune tolerance breakdown. The presentation of citrullinated peptides to T-cells and the subsequent activation of B-cells with production of ACPAs trigger an inflammatory process characterized by cellular infiltration and release of specific cytokines, chemokines and growth factors [39]. Specifically, cytokines such as IL-4, IL-13 and TGF-β, and growth factors such as platelets-derived growth factor (PDGF) promote fibroblast differentiation and proliferation, providing a potential link between inflammation and fibrosis. Matrix metalloproteinases (MMPs) contribute to the crosstalk between inflammation and tissue remodelling pathways; in fact, MMPs are activated in damaged epithelia and promote cellular recruitment and activation of cytokines and pro-fibrotic mediators. Angiogenesis plays also a role in this process, with direct links to both inflammation and fibrosis [42].

**Fig. 3:**
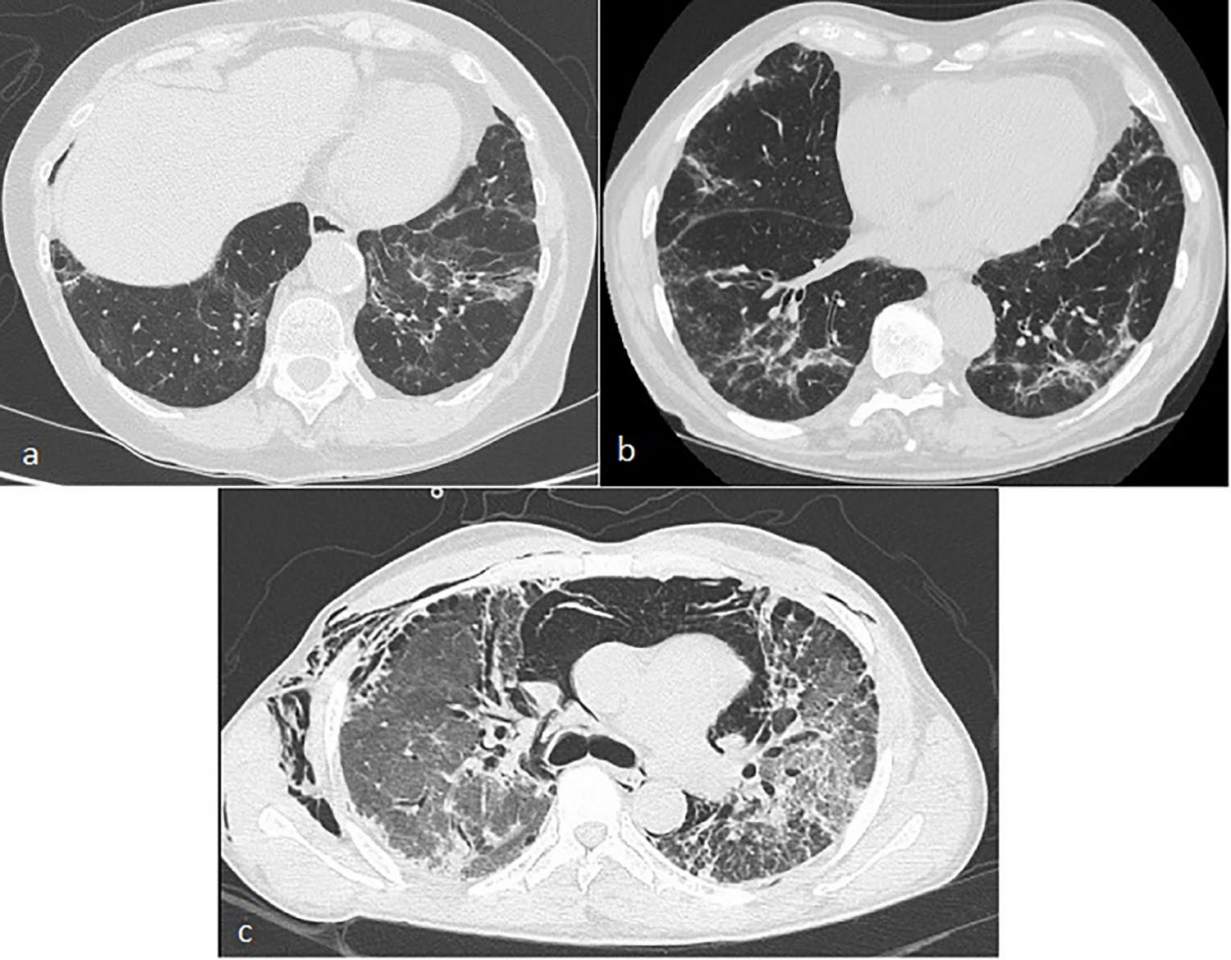
HRCT images. **a**: Fibrosing OP in a patient with dermatomyositis; **b**: NSIP pattern in a patient with anti-synthetase syndrome (Anti-Jo1 +); **c**: rapidly progressive ILD complicated by pneumomediastinum and subcutaneous emphysema in a patient with Clinically Amyopathic Dermatomyositis and anti-MDA5-antibodies positivity

In contrast to other rheumatic diseases, the predominant pattern of RA-ILD is UIP (Fig. 3), followed by NSIP. The clinical course of RA-ILD is highly variable and heterogeneous, ranging from asymptomatic to rapidly progressive disease. UIP pattern in RA is associated with poor prognosis, in particular more frequent hospitalization and need for oxygen therapy, rapid decline of pulmonary function and worse survival compared to non-UIP pattern [43]. Notably, RA-ILD patients with the UIP pattern can experience episodes of acute exacerbations similar to those reported in patients with idiopathic pulmonary fibrosis (IPF) [44]. Among other variables associated with mortality, older age was a significant predictor of poor prognosis in several studies [34]–[36]. Other factors include: male sex, ILD severity as assessed by reduced DL_CO_ and FVC, extent of fibrosis on HRCT, acute exacerbations of RA-ILD and RA disease activity [3]. Bronchoalveolar lavage (BAL) fluid cytology shows non-specific findings, such as an increased neutrophil count in patients with UIP, and a predominantly lymphocytic cytology in patients with NSIP or organizing pneumonia. Importantly, these features are not predictive of RA-ILD progression and response to treatment [3].

### Idiopathic Inflammatory Myopathies

In the majority of cases, myositis-associated lung involvement manifests as ILD, with a prevalence ranging from 23 to 65% based on the diagnostic tools used [7]. Substantial heterogeneity exists within the spectrum of IIMs, and each condition is associated with various frequencies of ILD. Such heterogeneity might be explained, at least in part, by the presence of various autoantibodies. In particular, myositis-specific autoantibodies (MSAs), such as the anti-aminoacyl-tRNA-synthetase (ARS) antibodies and anti-melanoma differentiation factor 5 (MDA5), are associated with a high prevalence of ILD, which can precede, follow or be concomitant with the onset of skin or muscle manifestations, but tends to appear early in the disease course [45].

The pathogenesis of lung involvement in IIMs is poorly understood. However, similar to RA-ILD, some evidence suggests that—at least for the subsets with high prevalence of lung involvement—the lung might be the initiating disease site, being repeatedly exposed to environmental immunogenic stimuli (e.g. pollutants, infectious agents or cigarette smoke, among others) [46, 47]. These stimuli induce inflammation and cellular distress or death, leading to enhanced release of microparticles with aberrant self-antigen exposure. Subsequently, a break of immune self-tolerance occurs, with migration of inflammatory cells to the lung (Fig. 1). In particular, ARS antibodies may exert a chemokine-like activity, inducing migration of lymphocytes and activated monocytes [46, 48]. At the site of inflammation, dendritic cells present the antigen/s to T cells promoting their proliferation via a major histocompatibility complex (MHC)-II-dependent process. The role of T cells in disease pathogenesis is corroborated by the presence of CD3+ lymphocytes in lung infiltrates of patients with polymyositis and ILD. In addition, CD3+ lymphocytes display a restricted T-cell receptor repertoire in their variable region, suggesting an antigen-driven oligoclonal lung infiltration [49]. Gono et al. found significantly increased serum levels of inflammatory cytokines such as IL-6, IL-8, tumour necrosis factor-alpha (TNF-α) and interferon gamma-induced protein 10 in patients with IIMs-ILD compared to IIMs patients without ILD [50]. In addition, Eloranta et al. showed that sera from patients with IIMs-ILD have significantly higher IFN-α inducing activity compared to sera of patients without ILD. This association was not observed for other manifestations of IIMs suggesting a specific role for IFN-a in the maintenance of lung inflammation [51]. Natural killer (NK) cells are thought to contribute to protein cleavage and generation of self-antigens by producing granzyme B. In this regard, massive infiltrates of NK cells are found in the lungs of anti-Jo1-positive patients in histological studies [52].

The clinical course of IIMs-ILD ranges from chronic forms with slowly progressive symptoms to acute and rapidly progressive forms leading to respiratory failure—typical of patients with clinically amyopathic dermatomyositis (CADM) and anti-MDA5 antibodies, occasionally associated with the development of pneumomediastinum (Fig. 3) [53, 54]. In a significant proportion of IIMs patients, ILD can also be asymptomatic/subclinical, whereas in other cases PFTs show a restrictive ventilatory defect with a reduced DL_CO_ [45]. It is important to underline that the interpretation of PFTs in patients with IIMs may be influenced by extra-parenchymal factors, such as inspiratory muscle weakness that can result in reduced FVC. In some cases, muscle strength can improve with treatment [27, 45]. NSIP is the predominant radiological and histological pattern followed by UIP; other histological patterns observed in patients with IIMs-ILD include organizing pneumonia (OP) and diffuse alveolar damage (DAD) [7, 55]. In rare cases of anti-synthetase antibodies positivity, OP is associated with bronchiolar involvement (BOOP, bronchiolitis obliterans with organizing pneumonia) [56].

### Sjögren’s Syndrome

In patients with SS, the prevalence of ILD is around 20% when defined by radiological (i.e. HRCT) or functional (reduced FVC and/or DL_CO_) criteria [57]. It mainly develops after SS onset, with its prevalence increasing with disease duration [57]. While radiographic abnormalities on HRCT are common, the prevalence of clinically significant pulmonary disease is around 9–12% [58]. Many patients are asymptomatic and lung involvement is generally mild and slowly progressive. Although lymphocytic interstitial pneumonia (LIP) has been classically linked to SS, more recent studies showed that NSIP is the most common histological pattern, followed by UIP, LIP and OP [55]. SS is characterized by lymphocytic infiltration and marked B cells hyperreactivity. Rarely, primary pulmonary lymphoma and amyloidosis are found [58].

### Systemic Lupus Erythematosus

Parenchymal involvement is a rare complication of systemic lupus erythematosus (SLE), occurring in approximately 10% of patients [4, 57]. Tissue injury in SLE is the consequence of several factors: autoreactive dendritic cells stimulate CD4+ T-cells that infiltrate target tissues and induce the activation of B cells, leading to autoantibodies production [59]. NSIP is the most common histopathologic pattern, although LIP, OP and UIP can also be found. In most cases, clinical progression is slow and lung function stabilizes over time [60]. Some patients (from < 2% to 5,4%) may present acutely with severe respiratory failure secondary to diffuse alveolar haemorrhage (DAH), which is associated with a high mortality (50%) [55, 61]. The most relevant risk factor for the development of DAH is serologically and clinically active disease, in particular with class III or IV lupus nephritis [57]. In these cases, DL_CO_ is typically increased due to the presence of haemoglobin within the alveolar spaces [57].

## Airways

Airway involvement is common in patients with RA, but is seldom reported in patients with other ARDs.

### Rheumatoid Arthritis

Airway involvement is observed in 39–60% of patients with RA, when assessed by HRCT. Both upper and lower airways can be involved [7, 62]. Cricoarytenoid joint (CJ) arthritis is the most common form of upper airways involvement and is generally clinically silent [63]. CJ arthritis is the consequence of synovial fluid accumulation in the CJ capsule, leading to erosion and subluxation of the cartilage that may ultimately result in fixation [63]. With regard to lower airways, RA has been associated with airway hyperresponsiveness and small airway obstruction (30–73% of patients) [62], and with high prevalence of HRCT abnormalities, such as bronchial wall thickening and bronchiectasis (BE), which are observed in as many as 30% of patients with RA without ILD [62, 63]. The genetic predisposition to ILD and airway disease appears to be different, with carriage of the HLA-DRB1*1501 and *1502 allele exerting opposite effects as a risk factor for ILD and protective factor against airway disease, respectively [64]. Similar to RA-ILD, airway disease can be found in preclinical RA, whereas in patients with clinically overt RA, airway disease is associated with higher disease activity and autoantibody positivity (RF and ACPAs), even when smoking status is taken into account [7, 65]. The mechanisms of coexistence of RA and BE is unclear. A possible hypothesis is that chronic bacterial infection secondary to BE acts as a prolonged antigenic stimulus that leads to breakdown of immune tolerance and subsequent development of RA in genetically predisposed individuals [7, 66]. Two forms of small airways involvement have been described: follicular bronchiolitis (FB) and constrictive bronchiolitis. FB is characterized by hyperplasia of bronchial-associated lymphoid tissue (BALT), whereas constrictive or obliterative bronchiolitis is characterized by peribronchiolar inflammation and fibrosis without evidence of lymphoid hyperplasia [63, 67].

### Sjögren’s Syndrome

Airway disease is the most common manifestation of respiratory involvement in SS. Airway abnormalities may involve the trachea, bronchi and bronchioles and can be related to either sicca syndrome (glandular involvement) or cell infiltrate (extra glandular involvement) [58]. Tissue infiltrate typically contains CD4+ T lymphocytes [68]. Large airways involvement, as confirmed by pulmonary function testing, affects 8 to 12% of SS patients [58]. Xerotrachea manifests as dryness of the mucosa of the trachea and is characterized by decreased mucociliary clearance, due to local glandular involvement [58]. Bronchiolitis is the most frequent form of airway disease and may be isolated or associated with ILD [68]. The most frequent form of bronchiolitis is FB, which is characterized by hyperplastic lymphoid follicles with reactive germinal centres along the bronchovascular bundle [58, 69]. FB results from antigenic stimulation of the BALT with polyclonal lymphoid hyperplasia [69]. Less frequently, bronchiolar involvement might present as BOOP [56]. In SS, the prevalence of BE ranges from 7 to 54%; they are generally cylindrical and mainly located in the lower lobes [69]. Patients with BE tend to be older at SS diagnosis and display higher frequency of hiatal hernia, lower prevalence of anti-Ro/SSA antibodies and increased prevalence of anti-smooth muscle cell antibodies [57].

### Systemic Lupus Erythematosus

Airway disease is an uncommon manifestation of SLE, and both upper and lower airways may be involved. Upper airway disease is rare and ranges from mild ulceration to vocal cord paralysis, cricoarytenoid arthritis and necrotizing vasculitis with airway obstruction [61]. Bronchial wall thickening and BE are detected on chest HRCT in approximately 20% of SLE patients, but they are commonly clinically silent [61]. About 25% of patients show signs of small airways dysfunction at PFTs, although bronchiolar abnormalities on HRCT are uncommon [70]. Cases of lupus pneumonitis presenting with focal bronchiolar lymphocytic inflammation causing an acute obliteration of small airways with intraluminal fibroblastic plugs consistent with BOOP have also been reported [71, 72]. This entity is rare in SLE patients, but it is associated with high mortality rates and poor response to immunosuppressants and Glucocorticoids (GCs) [73].

### Systemic Sclerosis

In SSc, airway involvement is rare compared to other ARDs [74]. Although autoptic studies of SSc lungs showed atrophic and fibrotic lesions in muscular, elastic and adventitial layers of bronchial wall, a definite obstructive pattern on PFTs is uncommon in SSc [75]. Around 20% of patients present small airways dysfunction even in the absence of ILD [76]. In a recent study on non-smoker SSc patients, markers of small airways stiffness (of both resistance and reactance) were higher than in age-matched healthy controls and associated with ground glass opacities or reticulation on HRCTs [77]. However, changes in resistance and/or reactance were not predictive of lung function deterioration at 12 months [77].

## Pleura

Pleural involvement is common in SLE and to a lesser extent in RA, but is a rare finding in other ARDs.

### Systemic Lupus Erythematosus

Pleural effusion is the most common pulmonary manifestation of SLE. Clinically relevant pleural effusion is observed in up to 50% of SLE patients during the disease course, with effusions being either bilateral or unilateral [7, 57]. The pathogenesis of lupus pleuritis involves immune complexes (IC) deposition from systemic circulation with activation of complement and infiltration of inflammatory cells [78]. Long-standing disease, late-onset SLE, positive anti-RNP and anti-Smith antibodies are associated with a nearly twofold increased risk of developing pleuritis [57]. In addition, pleural involvement is more common in patients with active disease and multiorgan involvement [57]. Pleural fluid in lupus pleuritis is often exudative with increased leukocyte (i.e. neutrophils or lymphocytes) cell count [7]. In patients without a previous diagnosis of SLE, the presence of high titres of antinuclear antibodies (ANA) (i.e. > 1:160) in the pleural fluid supports the diagnosis of lupus pleuritis [78], although this phenomenon can also be observed in malignancies [57]. When performed, pleural biopsies reveal non-specific findings, namely lymphocytic and plasma cell infiltration, fibrosis and fibrinous pleuritis.

### Rheumatoid Arthritis

Pleural effusion in RA is found in up to 20% of patients and is more frequent in middle-aged (> 50 years) males, particularly in patients with long-standing arthritis, smoking history and underlying lung disease [6]. Rheumatoid effusion is usually exudative with low glucose levels and unilateral (generally affecting the left side) in the majority of patients (around 70%) [5]. The levels of RF in rheumatoid pleural effusion are generally similar or higher than those in the serum, with the presence of RF in the pleural effusion being strongly suggestive of a rheumatoid origin [5]. Patients with rheumatoid effusions display also high levels of pleural sC5b-9 (a product of alternate pathway complement system activation) and low levels of C3, C4, indicating a local autoimmune activation [79].

### Systemic Sclerosis

In SSc, pleural involvement is rarer than pericardial disease, affecting approximately 5% of patients and more frequently those with a diffuse cutaneous form [74, 80]. Pleural effusion consists mainly of lymphocytes or eosinophils, with one study reporting elevated levels of CA125 in both serum and pleural fluid [81]. The pathogenesis of SSc “pleuropathy” is poorly understood, but it has been suggested that microvascular injuries (driven by anti-endothelial autoantibodies) may increase capillary permeability causing the exudation of fluids and white cells in the pleural space [82].

### Sjögren’s Syndrome

Pleural involvement is unusual in SS, being reported in only 1% of patients [83, 84]. Similar to SSc, pleural fluid analysis usually shows a lymphocytic exudate, with no specific feature [6]. The pathogenesis of SS pleuropathy is unknown and may differ from patient to patient, reflecting the pleiotropic course of the disease.

## Pulmonary Vasculature

The typical presentation of pulmonary vascular involvement in ARDs is pulmonary arterial hypertension (PAH), which occurs mostly in patients with SSc and less frequently in SLE [8].

### Systemic Sclerosis

PAH is detectable in about 8–12% of SSc patients, being more common in those with limited cutaneous disease, and associated with anticentromere positivity [8]. Unlike ILD, PAH is rarely the presenting manifestation of the disease, as it often occurs 10–15 years after the diagnosis of SSc [7].

Endothelial dysfunction plays a key role in the pathogenesis of SSc-PAH. It is characterized by an imbalance between vasoactive, proliferative mediators, primarily endothelin-1 and thromboxane A2, and vasodilators (e.g. nitric oxide, prostacyclin) and antiproliferative mediators. Endothelin-1 is overexpressed in plasma and lung tissue of patients with SSc-PAH and SLE-PAH [8]. Moreover, a study from Becker et al. showed that the agonistic endothelin-1 type A receptor antibodies (anti-ETAR) are more common in SSc-PAH/ARDs-PAH than in other forms of PAH and may predict prognosis in SSc-PAH [85]. Endothelial dysfunction leads to changes in the vascular tone and promotion of vascular remodelling of small to medium‐sized pulmonary vessels [8, 86]. Pulmonary artery vasoconstriction is amplified by the release of serotonin by activated platelets, leading to hypoxemia and ischaemia reperfusion injury. In turn, this results in additional cytokine release, vascular remodelling, fibrosis and intraluminal microthrombosis. Consequently, a progressive increase in pulmonary vascular resistance, pulmonary artery pressure and right ventricular pressure overload occurs, leading to right heart failure. Inflammation is believed to play only a marginal role in SSc-PAH, although inflammatory cells have been hypothesized to induce a maladaptive right ventricular remodelling [9, 87].

Several forms of pulmonary hypertension (PH) can occur in SSc: isolated PAH (group I according to the 2015 ESC/ESR guidelines), PH secondary to left heart dysfunction (group II) and PH secondary to ILD or hypoxia (group III) [88]. In patients with diffuse cutaneous form, PH is most frequently secondary to left heart disease and ILD [89]. Nevertheless, in SSc different forms of PH may occur in the same patient, making diagnosis and management of SSc-PH particularly challenging in some cases. Doppler transthoracic echocardiography is recommended as a screening test in all patients at SSc diagnosis, followed by annual screening with echocardiography, DL_CO_ and biomarkers (e.g. N-terminal pro-brain natriuretic peptide) according to the ESC/ESR 2015 guidelines and screening algorithms [88, 90].

### Systemic Lupus Erythematosus

The prevalence of SLE-PAH is about 5%, with a 1-year survival better than that of SSc-PAH [7]. The presence of Raynaud’s phenomenon, anti-RNP and anti-SSA/Ro antibodies are independent predictors of PAH development in SLE [91]. Particularly in SLE patients with antiphospholipid antibodies positivity, chronic thromboembolic PH (group IV) should also be included in the list of differential diagnoses with SLE-PAH.

Beyond the above mentioned mediators of endothelial dysfunction, macrophagic and lymphocytic infiltration as well as deposits of both immunoglobulins and complement have been shown in the wall of small pulmonary arteries in patients with SLE-PAH, supporting an important pathogenetic role of inflammation and autoimmunity. B cells contribute to pulmonary vascular injury and remodelling by producing autoantibodies that cause endothelial dysfunction, while cytotoxic T cells appear to play a role in arterial muscularization. [92]. Thus, both acute (e.g. fibrinoid necrosis and vasculitis) and chronic lesions (e.g. intimal and periadventitial fibrosis, thickening of the media) may be found in SLE-PAH. In this regard, the existence of two major phenotypes of PAH secondary to SLE has been postulated: a “vasculitic subtype” detectable in patients with high SLE disease activity (e.g. Systemic Lupus Eriyhematosus Disease Activity Index 2000, SLEDAI-2 K > 9, concomitant presence of rash, arthritis and nephritis) and a “vasculopatic subtype” characterized by a low activity of SLE and severe PAH [93]. This might also explain why, although the outcome is generally more favourable than in SSc-PAH patients, the prognosis of some patients with severe SLE-PAH is poor, with right heart failure and arrhythmias being the most common causes of death.

Notably, in patients with SLE as in those affected with other ARDs, echocardiography is recommended only in the presence of suggestive symptoms [88].

DAH is a rare but severe complication of SLE with a prevalence ranging between 2 and 5%, an overall mortality of approximately 50% and a high risk of recurrence [4]. The aetiology of this complication is not completely understood, but many patients develop DAH in association with lupus nephritis, suggesting a common immune complex (IC)-driven pathogenesis [94]. The deposition of IC in the alveolar capillaries is thought to be the initiating event leading to inflammatory cells infiltration or non-inflammatory bland haemorrhage. IC-induced capillaritis is characterized by a neutrophilic interstitial infiltration outside the capillaries [94]. Accumulated neutrophils undergo cytolysis with release of neutrophil extracellular traps (NETs) and cytotoxic proteins leading to loss of integrity of the alveolar–capillary basement membrane, resulting in leakage of red blood cells into the alveolar space. Non-inflammatory bland haemorrhage is associated with a predominantly monocytic infiltration in the alveolar wall and ICs deposition [94].

### Other ARDs

The typical vascular involvement of the lung in RA is rheumatoid vasculitis, characterized pathologically by destructive inflammatory infiltrate within small- and medium-sized blood vessel walls. PAH is rare in IIMs, SS and RA. In patients with RA, the prevalence of PAH was reported to be similar to that of the general population in a recent French national study [7, 95].

## Treatment

### Lung Parenchyma

When initially evaluating patients with ARDs-ILD, the first step is the identification of predictors of poor prognosis in order to stratify (and manage) patients based on the risk of ILD progression. In SSc patients with limited ILD extension according to Goh et al. (i.e. < 20% of lung parenchyma on HRCT) and without poor prognostic factors, a “watch-and-wait” strategy may be appropriate, as potential systemic side effects may outweigh the benefits of treatment [96]. However, timely intervention is of utmost importance in patients with severe or progressive disease to stabilize lung function or slow disease progression and prevent irreversible damage. The decision to start treatment is therefore based on several factors that include severity of ILD based on imaging and PFTs (FVC and DL_CO_), rate of progression on serial PFTs and HRCT, likelihood of reversibility and duration of the underlying ARD. Individual factors should also be taken into account, including patient age, comorbidities and preference [97]. Specific treatment strategies may differ according to the underlying disease, although, with the exception of SSc, (GCs) represent the first-line treatment approach, followed by conventional immunosuppressants and biologic drugs in refractory cases [97, 98]. In this scenario, the role of new antifibrotic drugs such as nintedanib in the treatment of ARDs-ILD need to be determined. Therapies currently employed in ARDs-ILD may lead to lung function stability in many cases, with improvement being observed in a minority of patients, especially in SSc-ILD.

#### Systemic Sclerosis

In patients with subclinical ILD and low or no risk of disease progression, current approaches to treatment include routine clinical, functional and radiological follow-up. Conversely, patients with clinically significant ILD and those at risk of progressive ILD should be promptly treated with immunosuppressive agents [98].

Glucocorticoids are generally avoided at doses higher than 10–15 mg/daily, mainly due to the increased risk of renal crisis [97]. Despite its known toxicity, cyclophosphamide (CYC) should be considered for the treatment of severe SSc-ILD, particularly in rapidly progressive cases [98]. Indeed, the Scleroderma Lung Study I, a double-blind, randomized, placebo-controlled trial, showed that i.v. CYC improves FVC, total lung capacity (TLC), skin thickness, dyspnoea and quality of life after 12 months of treatment compared to placebo in patients with SSc-ILD [99]. However, while beneficial effects in most of these outcomes were still apparent several months after withdrawal of therapy, generally, they were no longer present 12 months after CYC discontinuation, underlying the need for a maintenance therapy [100]. In the Scleroderma Lung Study II, mycophenolate mofetil (MMF) 1.5 g twice daily was associated with an improvement in FVC after 24 months of treatment, comparable to CYC treatment for 12 months, but with a better safety profile, particularly with regard to the risk of cytopenia [101, 102]. In a observational study, azathioprine (AZA) following IV CYC induction therapy determined stability or improvement of lung function, with an efficacy similar to MMF and oral CYC [103]. Based on these results, the European League Against Rheumatism (EULAR) guidelines suggest CYC therapy be considered in SSc patients with progressive lung disease. Furthermore, tailoring of CYC dose and treatment duration should be determined individually, according to clinical presentation and treatment response [104]. Generally, CYC is administered for 6–12 months, followed by a switch to another agent, usually MMF, for maintenance therapy, [105].

Rituximab (RTX) may stabilize lung function, thus preventing further FVC and/or DL_CO_ decline [106–108]. In a monocentric study, the association of RTX and oral MMF, which is frequently used in clinical practice in severe ILD cases, showed a good efficacy and safety profile [109]. RTX is considered mainly in SSc-ILD cases refractory to conventional treatment, but recent data suggest RTX may be considered also as an early treatment option as a valid alternative to CYC [110]. In early diffuse SSc, tocilizumab (TCZ) treatment was associated with a lower proportion of patients experiencing a decline in FVC > 10% compared to placebo, although decline in lung function was not the primary endpoint of the study [111, 112]. In addition, subsequent analysis showed that TCZ was also associated with improvement of ILD compared to placebo, independent of the radiological extent of ILD [111]–[113]. Based on these data, the US Food and Drug Administration (FDA) approved tocilizumab for the treatment of SSc-ILD in March 2021. The evidence on the efficacy of abatacept (ABA), a soluble chimeric cytotoxic T- lymphocyte antigen 4 (CTLA4) protein, in SSc-ILD is limited: a multicentre double-blind randomized controlled trial showed no changes in FVC after 12 months of treatment in patients with diffuse cutaneous SSc [114]. Other small studies showed no effect on lung fibrosis or lung function in patients with SSc-ILD when ABA was prescribed for joint involvement [115, 116].

Based on preclinical evidence and clinical similarities between IPF and SSc-ILD, a phase III double-blinded, randomized, placebo-controlled study (SENSCIS trial) evaluated the safety and efficacy of nintedanib, an inhibitor of fibroblast growth factor receptors (FGFRs), platelet-derived growth factor receptors (PDGFRs) and vascular endothelial growth factor receptors (VEGFRs), for at least 52 weeks in patients with SSc-ILD [117]. Nintedanib treated patients experienced a statistically significant lower annual rate of decline in FVC compared with patients randomized to placebo (-52.4 mL/year vs. -93.3%, respectively) [117]. Nintedanib has been approved by the FDA and the European Medical Agency (EMA) for the treatment of SSc-ILD in September 2019 and April 2020, respectively. In addition, the most recent treatment algorithms in SSc-ILD suggest considering nintedanib both as a possible initial monotherapy and as a combination therapy in cases that progress despite immunosuppressants [118].

#### Rheumatoid Arthritis

The optimal treatment of RA-ILD has not been determined and is largely based on data derived from other ARDs-ILD, particularly SSc-ILD [3].

Glucocorticoids represent the first-line treatment, generally with oral prednisone at a daily dose of 0.5 mg/kg, with gradual tapering over weeks to months based on clinical response [3]. An immunosuppressive agent such as MMF or AZA may be added in patients who fail to respond to or experience intolerable side effects from corticosteroid treatment [3]. The safety and efficacy of MMF have been examined in a large group of patients with ARDs-ILD, including 18 patients with RA-ILD, treated with MMF for a median of 897 days. Overall, treatment with MMF was associated with either stable or improved pulmonary function. Among patients with RA-ILD, FVC trended downward prior to MMF initiation and upward following MMF treatment [119].

Observational and retrospective studies show that RTX may stabilize or even improve lung function over a prolonged follow-up period [120, 121]. TCZ may be used for treatment of patients with RA not responsive to Disease modifying anti-rheumatic drugs (DMARDs) to reduce disease activity and improve clinical, radiological, functional and patient-reported outcomes [97]. However, the effect of TCZ on the natural history of RA-ILD remains unclear [97]. An observational, multicentre study including RA-ILD patients showed a stabilization of lung function with ABA after a median follow-up of 12 months [122]. A significant number of patients with RA (n = 89) were enrolled in the INBUILD trial, which evaluated the efficacy of nintedanib vs. placebo in ILD patients with a progressive phenotype (i.e. functional and/or radiological progression or symptoms worsening within the previous 24 months) despite optimal treatment [11, 123]. Over the 52-week study duration, nintedanib as compared to placebo reduced the annual rate of FVC decline in patients with chronic progressive fibrosing ILD, irrespective of the underlying ILD diagnosis [123].

#### Idiopathic Inflammatory Myositis

No evidence-based guidelines exist regarding treatment of IIMs-ILD [98]. Since clinical features, treatment response and prognosis are highly variable among IIMs patients, the initial treatment should be based on ILD severity and mode of presentation [98]. For instance, amyopathic dermatomyositis with anti-MDA5 positivity is associated with rapidly progressive ILD, GCs resistance and poor prognosis [98]. Conversely, anti-synthetase syndrome (with ARS antibodies positivity) is associated with good response to GCs [98]. GCs are generally the first-line treatment, especially in patients with mild disease or chronic presentation [98, 124]. Other immunosuppressants are generally added in patients who fail to respond to GCs or suffer significant side effects [98, 124]. Observational studies have shown that MMF may improve or stabilize ILD in patients with IIMs [125, 126]. A retrospective study in patients with steroid-resistant ILD showed that treatment with CYC, AZA or MMF was associated with stabilization of pulmonary function, improved dyspnoea and reduction of steroid dose [126]. RTX appeared to be effective as a rescue therapy in patients with IIMs-ILD and progressive disease despite conventional therapy, including patients with anti-synthetase syndrome [127, 128]. Rapidly progressive forms, including those MDA5-related, are usually treated with high doses of GCs (i.e. 1 g/die for 3 consecutive days), CYC, calcineurin inhibitors or RTX in case of disease refractory to conventional therapies [45, 98]. A multicentre prospective study showed that a combined immunosuppressive regimen with high-dose GCs, tacrolimus and intravenous CYC, determined an improvement in 6-month survival rates in patients with MDA5 positive DM [129]. In addition, over a period of 52 weeks, improvements in anti-MDA-5 titers, serum ferritin levels, vital capacity and chest HRCT scores were observed [129]. Plasmapheresis (PEx) was used as additional treatment in cases who failed to respond to combination therapy (usually those presenting with low lymphocyte count, high ferritin levels and hypoxemia). A subsequent retrospective study demonstrated a better survival in patients treated with PEx in addition to such combination therapy, suggesting that PEx is an effective adjuvant treatment in anti-MDA5-positive DM with rapidly progressive ILD [130].

In non-randomized retrospective studies, tacrolimus, a calcineurin inhibitor, has been shown to improve or stabilize FVC and DL_CO_ in patients with progressive or refractory ARDs-ILD, especially polymyositis and dermatomyositis; in addition, it may lead to reduction and sometimes complete discontinuation of concomitant steroid/immunosuppressive treatments [131, 132]. A randomized controlled pilot 24-week trial, the ATtackMy-ILD trial, which is currently ongoing, will investigate the efficacy and safety of subcutaneous ABA compared with placebo in patients with anti-synthetase syndrome and ILD [98].

#### LES and Sjoögren’s Syndrome

No evidence-based guidelines for treatment of SLE-ILD or SS-ILD are currently available. The first-line regimen typically includes GCs, alone or in association with other immunosuppressive agents such as CYC or MMF [133]. RTX has been suggested as a second-line therapy [133]. In DAH, the most frequently used therapies are high-dose i.v. GCs (particularly methylprednisolone), CYC and plasmapheresis, although the latter does not appear to influence outcome [134]. Conversely, CYC was associated with improved survival [7, 94, 134]. RTX, in combination with GCs, may also be beneficial, though this has only been shown in case reports [57]. In SS, GCs, either alone or in combination with AZA or other immunosuppressants, are the treatment of choice [98]. In refractory cases, RTX should be considered [98].

#### Other thoracic involvements

The treatment of other thoracic manifestations of ARDs is not standardized, due to the paucity of sufficiently large case series or randomized clinical trials. Treatment of thoracic complications other than ILD is summarized in Table 1.

**Table 1:** Treatment of other thoracic manifestation of ARDs

Thoracic manifestations of ARDs other than ILD		
Airway involvement	Upper airways	Laryngeal involvement usually responds to oral GCs [135]
	Follicular bronchiolitis and obliterative bronchiolitis	Large series or randomized controlled trials are lacking. Some case series showed response to GCs alone or in association with immunosuppressants such as AZA or CYC [136]
Pleural involvement	- In mild cases of lupus pleuritis, GCs and hydroxychloroquine represent the first-line treatment. More severe cases require immunosuppressants such as MTX or biologic drugs such as the anti-Blys monoclonal antibody Belimumab. AZA, MMF, RTX, intravenous immunoglobulin and ciclosporin are used only in severe and refractory cases. In very rare cases of chronic symptomatic pleural effusion refractory to pharmacological therapy, pleurodesis or pleurectomy has been performed [34, 60] - In RA-associated pleuritis, specific treatment is usually not necessary	
DAD e DAH	- In SLE, the most common therapies are high-dose i.v. steroids (in particular methylprednisolone) in combination with immunosuppressive agents (such as CYC), plasmapheresis or intravenous immunoglobulins. Treatment with CYC showed an improved survival [7, 94] - Some case series suggest a potential role for RTX [94] - The use of methylprednisolone is recommended until cessation of haemorrhage [94]	
Pulmonary arterial hypertension	- Treatment of patients with ARDs-PAH should follow the same treatment algorithm as IPAH [90] - Several endothelin receptor antagonists (ambrisentan, bosentan and macitentan), PDE-5 inhibitors (sildenafil, tadalafil) and riociguat have been approved for treatment of ARD-PAH [104] - Continuous intravenous epoprostenol is considered the first-line agent in severe PAH (class III and IV). Other prostacyclin analogues (treprostinil, selexipag) have also been approved for treatment of PAH associated with ARDs [104] - In SLE-PAH, several observational cohort studies reported the efficacy of immunosuppressive therapies (mainly i.v. CYC or MMF and pulse prednisone) [88, 137]. Some studies also reported the use of GCs either alone or in association with standard vasodilators for treatment of milder SLE-PAH cases [138] - Immunosuppressive treatments failed to show efficacy in SSc-PAH [139]	

*SLE* Systemic Lupus Erythematosus, *PAH* Pulmonary Arterial Hypertension. *ARD-PAH* Autoimmune Rheumatic Diseases-associated Pulmonary Arterial Hypertension, *IPAH* Idiopathic Pulmonary Arterial Hypertension, *DAD* Diffuse Alveolar Damage, *DAH* Diffuse Alveolar Haemorrhage, *AZA* Azathioprine, *MTX* Methotrexate, *MMF* Mycophenolate mofetil, *CYC* Cyclophosphamide, *RTX* Rituximab, *PDE-5* phosphodiesterase type 5, *IV* Intravenous therapy, *GCs* Glucocorticoids

## Conclusions

Autoimmune rheumatic diseases exhibit various patterns of thoracic involvement in terms of prevalence, organ involvement, severity, progression and response to treatment, ranging from subclinical/mild involvement to rapidly progressive forms. This heterogeneity in presentation and clinical course is likely to reflect differences in the underlying pathogenetic mechanisms, although immune system dysregulation and fibrosis are shared pathogenic mechanisms. Therefore, it is difficult to establish a uniform treatment strategy for thoracic involvement in ARDs, particularly for ARDs-ILD, which is the most frequent manifestation. Although traditional immunosuppressive drugs (e.g. cyclophosphamide, mycophenolate mofetil) remain the mainstay of treatment, in the last few years, new biologic and non-biologic DMARDs have demonstrated some benefits. In particular, anti-fibrotic treatment (i.e. nintedanib) has proven efficacious in reducing functional decline and disease progression in ARDs-ILD with a progressive phenotype despite optimal treatment, irrespective of the underlying rheumatic disease. Nevertheless, a better understanding of disease pathogenesis remains essential for both the development of more targeted therapies and the optimization of available treatments.
